# Our Contemporaries

**Published:** 1915-03

**Authors:** 


### OUR CONTEMPORARIES.

**The Medical Times,** of January, in an article entitled "Some Thoughts on the Geography of Medical Greatness" grinds Baltimore, Boston and New York on the methods employed by the respective medical men to keep within the spot-light. Greatness seems to depend largely on little things, not only in the sense of careful attention to details in the line of duty but of taking petty advantages whenever they are presented. It is all right to jest about greatness, provided the jest leaves no sting but it strikes us as going a little too far to mention the publicity value of popular lectures on morality and to accuse the lecturer of having had clinical experience with his subject.

Editorially (ibid.) the opinion is expressed that sexual continence may be injurious to some persons.

**The American Practitioner,** November, 1914, contains a very sensible editorial on the ambiguities and fallacies of the Mann act. The sooner this country abandons the idea that drunkenness, sexual immorality and every other evil can be done away with by law and returns to the old ideal of personal liberty, the better off we shall be—not because liberty is preferable to good conduct but because strength of character can develop only in those who are free to choose between right and wrong.

**The American Practitioner,** now in its 49th volume and during the last few years, ably conducted by Dr. J. W. Wainwright, has been merged with the American Journal of Urology, Venereal and Sexual Diseases, edited and published by Dr. Wm. J. Robinson, 12 Mt. Morris Park, West, N. Y.

**The Critic and Guide,** February, 1915, emphasizes the expense and waste of too much system. In our experience, system consists in keeping everything of a kind together, applying the same principle to things used together. For example, arrange your reagents for urine, faeces, etc., in trays according to combined use; put all articles for repairing tires together, if possible. Unless your practice is highly specialized and you are doing much writing, make one record serve all purposes, financial and scientific. An elaborate card-index system may make you a slave and it is worse than useless unless rigidly maintained. Some years ago, we were asked to buy a roll-top desk with a complicated assortment of envelopes and cards to serve the purpose of a small envelope file and a couple of books compactly stored in about a tenth of the shelf space of the desk that we were—and are still—using.

---

**The Critic and Guide,** ibid., calls attention to the injustice of society in forbidding unmarried women with strong maternal instincts from having children. We confess to being hampered by conventionality in this respect but venture to suggest that there are plenty of children in orphan asylums that might partially solve the problem.

---

**The N. Y. World,** January 23, 1915, denounces the far-fetched application of the Mann Law, carried by a popular sentiment against literal white slavery, so as to cover immorality in general. "One single case of such juggling will do greater harm to the moral fibre of the community than a—(mentioning a man recently indited)—can do in a life-time."

